# Neutrophil-vascular interactions drive myeloperoxidase accumulation in the brain in Alzheimer’s disease

**DOI:** 10.1186/s40478-022-01347-2

**Published:** 2022-03-24

**Authors:** Leon C. D. Smyth, Helen C. Murray, Madison Hill, Eve van Leeuwen, Blake Highet, Nicholas J. Magon, Mahyar Osanlouy, Sophie N. Mathiesen, Bruce Mockett, Malvindar K. Singh-Bains, Vanessa K. Morris, Andrew N. Clarkson, Maurice A. Curtis, Wickliffe C. Abraham, Stephanie M. Hughes, Richard L. M. Faull, Anthony J. Kettle, Mike Dragunow, Mark B. Hampton

**Affiliations:** 1 Centre for Free Radical Research, University of Otago, Christchurch, New Zealand; 2 Department of Pathology and Biomedical Science, University of Otago, PO Box 4345, Christchurch, 8140 New Zealand; 3 Department of Pathology and Immunology, Center for Brain Immunology and Glia, Washington University in St. Louis, Campus, Box 8118, St. Louis, MO USA; 4 Centre for Brain Research, Faculty of Medical and Health Sciences, University of Auckland, Auckland, New Zealand; 5 Department of Anatomy With Medical Imaging, Faculty of Medical and Health Sciences, University of Auckland, Auckland, New Zealand; 6 Auckland Bioengineering Institute, University of Auckland, Auckland, New Zealand; 7 Department of Psychology, University of Otago, Dunedin, New Zealand; 8 Department of Biochemistry, University of Otago, Dunedin, New Zealand; 9 School of Biological Science, University of Canterbury, Canterbury, New Zealand; 10 Department of Anatomy, University of Otago, Dunedin, New Zealand; 11 Department of Pharmacology and Clinical Pharmacology, Faculty of Medical and Health Sciences, University of Auckland, Auckland, New Zealand

**Keywords:** Alzheimer’s disease, Neutrophil, Neutrophil extracellular trap, Blood–brain barrier, Myeloperoxidase

## Abstract

**Introduction:**

Neutrophil accumulation is a well-established feature of Alzheimer’s disease (AD) and has been linked to cognitive impairment by modulating disease-relevant neuroinflammatory and vascular pathways. Neutrophils express high levels of the oxidant-generating enzyme myeloperoxidase (MPO), however there has been controversy regarding the cellular source and localisation of MPO in the AD brain.

**Materials and methods:**

We used immunostaining and immunoassays to quantify the accumulation of neutrophils in human AD tissue microarrays and in the brains of APP/PS1 mice. We also used multiplexed immunolabelling to define the presence of NETs in AD.

**Results:**

There was an increase in neutrophils in AD brains as well as in the murine APP/PS1 model of AD. Indeed, MPO expression was almost exclusively confined to S100A8-positive neutrophils in both human AD and murine APP/PS1 brains. The vascular localisation of neutrophils in both human AD and mouse models of AD was striking and driven by enhanced neutrophil adhesion to small vessels. We also observed rare infiltrating neutrophils and deposits of MPO around plaques. Citrullinated histone H3, a marker of neutrophil extracellular traps (NETs), was also detected in human AD cases at these sites, indicating the presence of extracellular MPO in the vasculature. Finally, there was a reduction in the endothelial glycocalyx in AD that may be responsible for non-productive neutrophil adhesion to the vasculature.

**Conclusion:**

Our report indicates that vascular changes may drive neutrophil adhesion and NETosis, and that neutrophil-derived MPO may lead to vascular oxidative stress and be a relevant therapeutic target in AD.

**Supplementary Information:**

The online version contains supplementary material available at 10.1186/s40478-022-01347-2.

## Background

Neutrophils are the most abundant circulating leukocyte, and they play a critical role as first responders to inflammation [1]. However, there is now a growing appreciation for the role that neutrophils play in chronic diseases [2]. Indeed, neutrophils may have differential roles in the contexts of acute and chronic inflammation [2]. Most evidence from acute inflammation suggests that they are beneficial, especially in containing infection, while in chronic sterile inflammation evidence suggests that off-target effects of neutrophil activation can contribute to tissue damage [2].

Alzheimer’s disease (AD) is a neurodegenerative disease characterised by the accumulation of amyloid plaques and tangles of hyperphosphorylated tau [3]. Interestingly, AD has a chronic neuroinflammatory component that drives neurodegeneration [4–6], and cerebrovascular inflammation is important in recruiting peripheral immune cells in other neuroinflammatory diseases [7, 8]. Neutrophil accumulation in AD has been well documented [9–12], but recent reports also indicate that neutrophils contribute to pathology and cognitive impairment in AD [9, 11–13], consistent with the idea that they play a deleterious role in chronic diseases [2]. Indeed, there is an amyloid-independent improvement in cognition in mouse AD models when neutrophils are depleted or when interactions between neutrophils and cell adhesion molecules in blood vessels are blocked [9, 11, 12]. There have been reports of both neutrophil infiltration [10, 11] and vascular localisation [12, 13], and recent studies have indicated that neutrophil plugging of capillaries reduces cerebral blood flow in both the APP/PS1 and 5XFAD models of AD to drive cognitive deficits [12, 13], although the localisation of neutrophils in human Alzheimer brains has not been analysed in any detail.

In order to infiltrate the brain, neutrophils must pass across the blood brain barrier (BBB), formed by brain endothelial cells. Under normal circumstances, circulating neutrophils flow through cerebral vessels. However, following an injury, inflammation triggers the expression of chemokines and adhesion molecules in cerebral vessels that recruit and promote the attachment of neutrophils to the vessel wall [14, 15]. Concurrently, the glycocalyx, a proteoglycan structure that prevents the interaction of surface molecules such as cell adhesion molecules, collapses, facilitating neutrophil attachment [16]. Once adhered to the vasculature, the interaction of endothelial membrane protrusions, containing multiple adhesion molecules, facilitate the migration of immune cells into the brain parenchyma [17].

The neutrophil oxidant-producing enzyme myeloperoxidase (MPO) has also been suggested to play a role in AD [9, 12, 18–21]. Adoptive transfer of bone marrow from *Mpo*^−/−^ to 5XFAD mice reduces neuroinflammation, indicating a role for MPO-derived oxidants in AD-associated neuroinflammation [9]. While there is a consensus that MPO abundance is increased in AD, there are conflicting reports regarding its localisation and cellular source. MPO has been reported to localise to amyloid plaques [18, 20] and tau tangles [19], while the brain cells that have been reported to produce MPO include neurons [19], astrocytes [20], and microglia [19], as well as neutrophils [11]. The localisation and source of MPO has a profound impact on the targets of MPO-derived oxidants [22], especially because the major oxidant produced, hypochlorous acid (HOCl), is extremely reactive and has a limited diffusion radius [23].

In neutrophils, MPO is contained within cytoplasmic granules, and degranulation must occur before the enzyme is fully active. One way of enabling MPO release is the formation of neutrophil extracellular traps (NETs) in which mixing of chromatin and granule contents occurs, before the NET is expelled [24]. Once released, NETs are powerful activators of the immune response, forming a DNA–protein matrix decorated with damage-associated molecular patterns such as S100 proteins [25–27]. While infectious agents trigger NET formation [28], they have also been reported in sterile inflammatory diseases, including atherosclerosis and gall-stone formation [24, 29, 30]. There have been preliminary reports of NETosis in AD [11], however the abundance and localisation of the NETs have not yet been characterised.

We set out to investigate the accumulation and localisation of neutrophils in AD, as well as the source and distribution of MPO. We confirmed that there is neutrophil accumulation in AD, and demonstrated using a panel of antibodies that MPO was almost exclusively localised with other neutrophil markers. Strikingly, neutrophils accumulated throughout the vasculature in the AD brain, but only limited infiltration observed. We also observed rare deposits of MPO outside the vasculature, which were associated with plaques. To test whether plaque-associated MPO was present as NETs, we used a panel of antibodies to stain for potential NETosis, and while we did observe specific NET labelling these were only found in the vasculature. We also observed that there was a loss of endothelial glycocalyx staining in the AD vasculature, which may drive enhanced neutrophil-vascular interactions in AD.

## Materials and methods

### Human tissue

All post-mortem human brain tissue used in this study was obtained from the Neurological Foundation of New Zealand Human Brain Bank in the Centre for Brain Research, University of Auckland. All protocols in this study were approved by the University of Auckland Human Participants Ethics Committee (2008/279 and 011,654), and all families provided informed consent. All cases were examined by an independent neuropathologist and were classified based on neurological abnormalities, or lack thereof in neurologically normal control cases (Additional file 1: Table S1). Tissue microarrays of AD and control tissue were generated from paraffin-embedded blocks as described previously [31, 32].

### Animal models

#### APP/PS1 transgenic mice

APP/PS1 transgenic mice on a C57BL/6 J background were bred at Otago University, New Zealand, from stock imported from the Jackson Laboratory ((APPswe,PSEN1dE9)85Dbo, MMRRC stock No: 34832-JAX). Non-litter matched transgenic (n = 12) and wild-type (n = 12) male mice were obtained by breeding hemizygous males with wild-type females. Animals were single group-housed in standard caging on a 12-h light/dark cycle, and food and water were available ad libitum. Ethical approval for the mouse work was obtained from the University of Otago Animal Ethics Committee (AUP-19–82). Mice were euthanised by transcardial perfusion with saline followed by 4% paraformaldehyde (PFA) at 4 and 12 months of age, and tissue fixed by perfusion in 4% paraformaldehyde (PFA) in 0.1 M phosphate buffer (PB) for 1 day, followed by 30% sucrose in PB for 2 days. In all experiments, male mice were used. Brains were stored in optimal cooling temperature mounting medium and were sectioned in the sagittal plane (40 μm thickness). Sections were stored in cryoprotectant solution containing 30% sucrose and 30% ethylene glycol in PB at – 20 °C for later use.

#### Stroke

All procedures were performed in accordance with the guidelines on the care and use of laboratory animals set out by the University of Otago, Animal Research Committee (AUP-19–160). Focal stroke was induced by photothrombosis in adult male C57BL/6 J mice (3–4 months, 27–30 g) as previously described [33–35]. Under isoflurane anaesthesia (4% induction, 2–2.5% maintenance in O_2_) mice were placed in a stereotactic frame (9000RR-B-U, KOPF; CA, USA), and buprenorphine hydrochloride (0.1 mL of a 0.5 mg/kg solution, Temgesic.) was administered subcutaneously as pre-emptive post-surgical pain relief. Following sterilisation of the skin using chlorhexidine (30% in 70% ethanol, Hibitane), the skull was exposed through a midline incision, cleared of connective tissue and dried. A cold light source (KL1500 LCD, Zeiss, Auckland, New Zealand) attached to a 40 × objective providing a 2-mm diameter illumination was positioned 1.5 mm lateral from bregma. Then, 0.2 mL of Rose Bengal (Sigma-Aldrich; 10 mg/mL in sterile saline) was administered i.p.. After 5 min, the brain was illuminated through the exposed intact skull for 15 min, while keeping body temperature at 37 °C using a heating pad. The skin was glued, and animals left in a cage placed on a heating pad until they had recovered before being returned to their home-cage. Sham surgery was performed in the exact same way, except saline was injected instead of Rose Bengal. Mice were housed in groups of three to five under standard conditions in individually ventilated cages (IVC: Tecniplast: maintained at 21 °C ± 2 °C and humidity of 50% ± 10%), on a reverse 12 h light/dark cycle (white lights off from 07:00–19:00) with ad libitum access to food and water. Following stroke or sham surgery, animals were euthanised 1-, 3-, and 14-days post stroke (n = 6), with sham controls taken at 1 and 14 days (n = 3). Brains were fixed and embedded in paraffin blocks for sectioning.

### Immunohistochemistry

#### Paraffin-embedded tissue

Formalin-fixed paraffin embedded sections (7 μm thickness) were dewaxed in xylene for at least 1 h, followed by rehydration through an alcohol series. Heat-induced epitope retrieval was performed in tris–EDTA (10 mM tris–HCl, 1 mM EDTA, 0.05% Tween-20 (v/v), pH 9.0) using a pressure cooker. For the detection of amyloid-β and phospho-tau, antigen retrieval was performed in 80% formic acid for 3 min. Once sections were cooled and washed, they were blocked in 10% normal donkey serum (NDS) for 1 h. Primary antibodies, diluted appropriately (in 1% NDS in phosphate-buffered saline (PBS)), were added to sections overnight at 4 °C (antibody details in table S2). Primaries were removed, and sections washed in PBS, before appropriate species-specific secondary antibodies with Hoechst 33,342 (50 μg/mL; ThermoFisher, OR, USA) or DAPI (500 ng/mL; ThermoFisher) were added (in 1% NDS in PBS) for 4 h at room temperature. Coverslips were mounted onto sections with Prolong Gold antifade mountant (ThermoFisher) before imaging. Large human sections were imaged on a Nikon Eclipse N*i* microscope (20 × objective, NA 0.50; Nikon, Japan) with large regions acquired by tiling. Mouse sections were acquired using a Zeiss Axio Imager (20X objective, NA 0.5; Carl Zeiss, Germany) with Apotome-based deconvolution.

#### Massively multiplexed immunostaining

Using a protocol adapted from Maric et al. [36] and Murray et al. [37], paraffin-embedded tissue microarray sections of AD and normal middle temporal gyrus were processed as above. Imaging was carried out with an automated fluorescence microscope (Zeiss Z2 Axioimager) equipped with MetaSystems VSlide slide scanner (MetaSystems) running MetaFer (V 3.12.1) with a 20 × air objective (0.9 NA). This microscope is equipped with 6 custom excitation/dichroic/emission filter sets optimised for spectral separation of compatible fluorophores as previously described (Maric et al. [36]). Antibodies were then stripped from sections with the addition of 5X NewBlot™ Nitro Stripping Buffer (Li-Cor, NE, USA) for 10 min at room temperature. Sections were then washed in PBS, epitope retrieval performed where necessary, and a subsequent round of immunostaining and imaging performed as above. This was completed over four rounds. Alignment of images from all four rounds was performed using a custom Python script [38]. We confirmed the effectiveness of stripping at removing previous antibodies in Additional file 1: Figure S5.

### Neutrophil isolation

Venous blood was collected from healthy donors with informed consent under the ethical approval of the Southern Health and Disability Ethics Committee (Wellington, New Zealand [URA/06/12/083]). Neutrophils were isolated as previously described [39]. Briefly, dextran (1% (w/v)) sedimentation was followed by Ficoll density centrifugation, then hypotonic lysis to remove remaining red blood cells. Granulocytes were suspended in RPMI-1640 (Gibco, CA, USA) containing 2% FBS plus 10 mM HEPES. Purity was assessed by flow cytometry, and cultures were routinely > 98% pure. Where relevant, serum was obtained from blood collected without anticoagulant and left to clot at room temperature. The clot was pelleted (1200×g; 2 min) and the serum was collected and stored on ice until required.

### Amyloid-β preparation

Lyophilised Aβ_1-42_ (H1368, Bachem) peptide was resuspended in 1,1,1,3,3,3-hexafluoro-2-propanol (HFIP; 105,228, Sigma) to 1 mM through the rubber septum using a 2.5 mL glass Hamilton syringe with a Teflon plunger and sharp needle and allowed to sit at room temperature for 30 min. Aβ_1-42_-HFIP solution was aliquoted into single-use lo-bind Eppendorf tubes and allowed to sit overnight in a fume hood to evaporate HFIP. To ensure complete removal, tubes were transferred to a SpeedVac and dried for 1 h at room temperature. The resulting peptide films were stored at – 20 °C until use. To generate a monomeric preparation of Aβ_1-42_ the peptide film was allowed to come to room temperature, diluted to 5 mM in DMSO and sonicated for 10 min in a bath sonicator at room temperature. Aβ_1-42_ was further diluted in phenol-red free DMEM (Gibco) to 100 μM and used immediately for the monomeric preparation. To generate oligomers, the monomeric preparation method was used, however, the resulting Aβ_1-42_ preparation was incubated at 4 °C for 24 h prior to use. To generate fibrils, the Aβ_1-42_ solution was diluted to 100 μM in 10 mM HCl and incubated at 37 °C for 24 h prior to use. To generate aggregates, Aβ_1-42_ was resuspended in sterile water at 500 µM, vortexed thoroughly, and stored in aliquots at – 20 °C. In all instances Aβ_1-42_ was diluted to a final concentration of 1 µM in culture media.

### ELISA

#### MPO

Myeloperoxidase abundance and activity was measured in clarified brain tissue lysates from the entorhinal cortex. Brain samples that had been snap frozen at -80 °C were placed in lysis buffer (25 mM Tris–HCl pH 7.5, 150 mM NaCl, 50 mM NaF, 0.5 mM EDTA pH 8, 0.5% Triton-X 100™, 5 mM β-glycerophosphate, with fresh 1 mM DTT, 1 mM PMSF, 1 mM Na3VO4) with 1 mm stainless steel beads and homogenised with a bead beater. The lysate was then spun at 14 000 × *g* for 20 min, and the supernatant containing soluble protein taken. Samples were normalised to protein content in diluent (1% (w/v) bovine serum albumin (BSA) in PBS with 0.05% Tween-20 (v/v)). A high-binding 96 well plate was then coated in a mouse anti-MPO capture antibody (1:500 in PBS; clone 4A4, ThermoFisher) overnight at 4 °C. The following day, wells were blocked in diluent for 1 h at room temperature. Samples were added alongside an 8-point standard curve (0.7 – 50 ng/mL) of purified MPO, and left for 1 h at room temperature. AmplexRed assay buffer (50 μM AmplexRed, ThermoFisher; in 50 mM phosphate buffer with 50 mM NaBr and 20 μM H_2_O_2_) was added for 15 min, and fluorescence (ex 530 nm, Em 590 nm) was measured on a Synergy Neo2 HTS plate reader (BioTek, VT, USA). A rabbit polyclonal antibody to MPO (1:800 in diluent) was then added overnight at 4 °C. Biotinylated goat anti-rabbit secondary antibody (1:2000; Sigma, MO, USA) was added for 1 h at 37 °C, followed by ExtrAvidin alkaline phosphatase (1:1000; Sigma) for 1 h at room temperature. The alkaline phosphatase activity of samples was then measured from the conversion of *p*-nitrophenyl phosphate, with absorbance measured at 405 nm using a VarioSkan™ LUX microplate reader (ThermoFisher).

#### Calprotectin

The abundance of calprotectin was measured in samples using the commercially available CALPRO ELISA kit (Svar, Sweden) as per manufacturer’s instructions.

### Sytox green NETosis assay

Acutely isolated neutrophils were seeded in RPMI-1640 with 2% FBS and 5 μM Sytox Green (ThermoFisher) at 100 000 cells/well in a 96 well plate. A control where Sytox Green was absent was included for background subtraction. Neutrophils stimulated with phorbol 12-myristate 13-acetate (20 nM, PMA; Sigma) were used as a positive control. Neutrophils were treated with amyloid-β_1-42_ (1 μM, Aβ_1-42_), with 10% (v/v) autologous donor serum, the monoclonal antibody against Aβ_1-42_ 4G8 (10 ng/mL), or vehicle (0.01% DMSO). Fluorescence (ex 460 nm, Em 516 nm) was measured every 5 min on a Synergy Neo2 HTS plate reader (BioTek, VT, USA).

### Thioflavin T assay

Freshly prepared Aβ_1-42_ species or vehicles were added to PBS containing 20 mM thioflavin T (ThT). Samples were left at 37 °C for 1 h, then fluorescence (ex 440 nm, em 492 nm) measured with a VarioSkan™ LUX microplate reader.

### Negative-stain transmission electron microscopy

Copper grids with 300 meshes coated with formvar/carbon film (ProSciTech, Australia) were floated on a 5 μl drop of a 50 µM Aβ1-42 protein sample and incubated for 60 s. Grids were washed once with water, and then floated on 5 μl of uranyl acetate solution (2% w/v) for 30 s. Micrographs were taken on a Philips CM200 200 kV transmission electron microscope equipped with a Gatan digital camera.

### Image analysis

All automated image analysis was performed on CellProfiler (v4.0.7) using custom pipelines. The experimenters were blinded to the genotype and pathology of the patients in all cases.

#### Neutrophil and NET abundance

Neutrophils were thresholded based on intense staining for MPO and S100A8 following rolling ball background subtraction and smoothing. Large objects were excluded. NETs were identified as being positive for CitH3, S100A8, and MPO. A lectin mask was used to determine their localisation as vascular.

#### Lectin intensity

A low all-vessel threshold was performed following rolling ball background subtraction to include mid and low intensity vessels. Alternatively, collagen IV-positive vessels were identified with a high threshold. Large and small objects were filtered out. The size of objects was measured and segregated into ‘large’ and ‘small’ vessels based on the minor axis length cut-off of 12 μm (lectin) or 15 μm (collagen IV). The mean intensity within the large vessel and small vessel objects was then measured.

#### MPO colocalisation/association

MPO, S100A8, amyloid-β, and pTau positive structures were thresholded following rolling ball background subtraction. Objects that were touching were considered colocalised/associated.

#### Vascular vs extravascular MPO

A low all-vessel threshold was performed as above and dilated, then the MPO image masked by this. MPO-positive objects were then identified in the masked area (vasculature) and the unmasked area (parenchyma).

### Yang et al***.*** database [40]

Single-nuclear RNA-seq counts from the human AD vasculature were obtained from https://twc-stanford.shinyapps.io/human_bbb/.

### Data analysis

Data were analysed with GraphPad Prism (v 9.01). Data from larger TMA cohorts are presented as box-and-whisker, with the range, interquartile range, and median presented. Where outlier values were detected (> 2 SD outside the mean), staining quality was inspected to ensure the validity of results and if artefacts were detected, the outlier was removed. Otherwise, the value was included. All other data are presented as mean ± standard error of mean. Appropriate statistical tests were carried out, following a test of the normality of data. Briefly, t-tests were performed where there were two groups, and two-way ANOVA where there were two factors. Multiple comparisons were performed with Tukey’s post-hoc test.

## Results

### Neutrophils accumulate in the human AD brain and APP/PS1 mouse model of AD

**Fig. 1:**
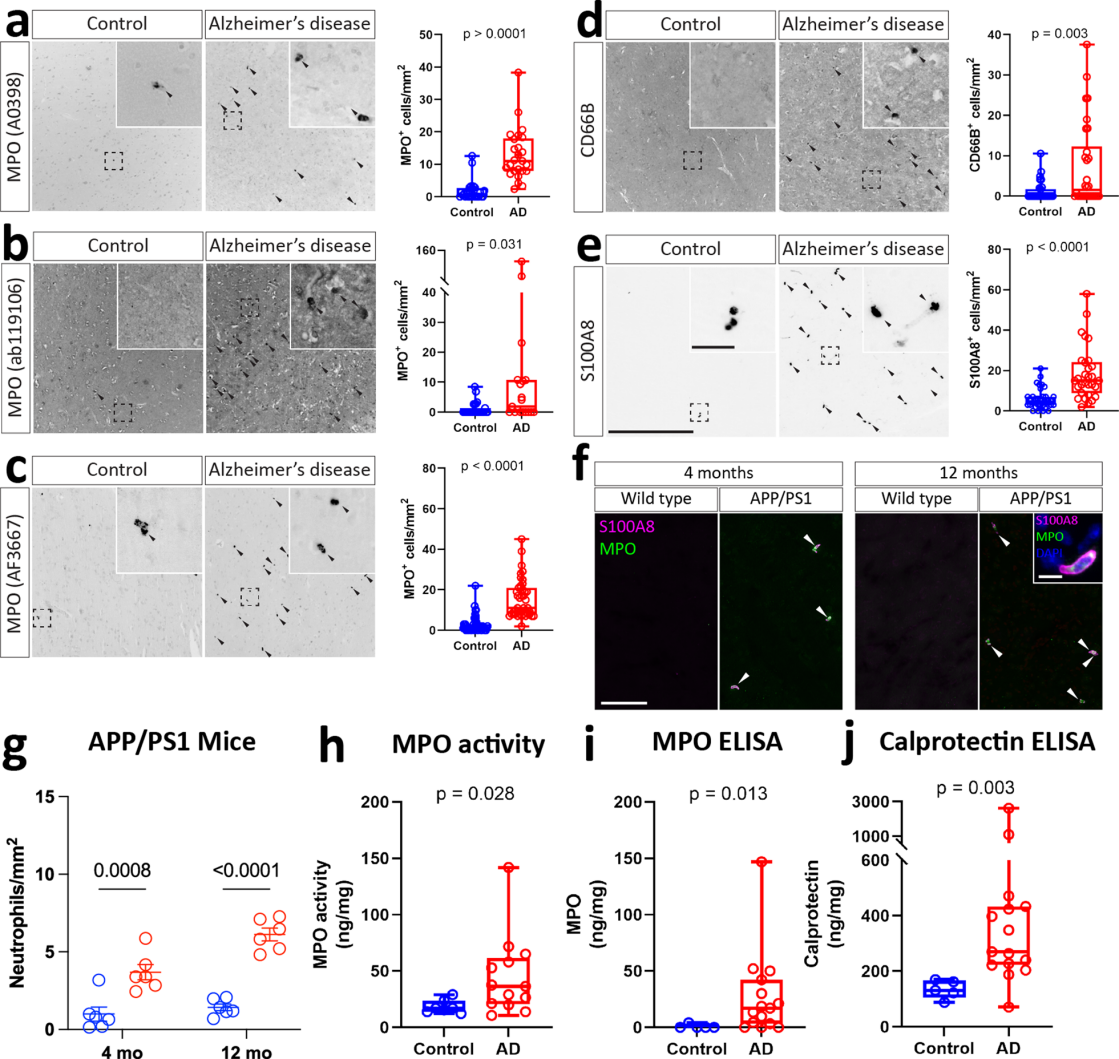
Cerebral accumulation of neutrophils in APP/PS1 mice and human Alzheimer’s disease. Human brain tissue microarrays (N = 21–46 per condition) of middle temporal gyrus were stained for neutrophil markers **a** MPO (A0398), **b** MPO (ab1191060), **c** MPO (AF3667), **d** CD66B, and **e** S100A8. Scale = 250 μm, inset = 25 μm. P-values represent results of an two-tailed Student’s T-test.Brain sections from APP/PS1 or wild type mice were taken at 4 and 12 months, and stained for the neutrophil markers S100A8 and MPO. **f** Representative images and **g** analysis of neutrophil abundance in mouse brains. Scale = 100 μm. P-values represent results of a two-way ANOVA. The abundance and activity of neutrophil markers **h**, **i** MPO and **j** calprotectin in human brain tissue lysates was measured by ELISA (N = 7 – 13 per condition). P-values represent results of an two-tailed Student’s T-test

We initially used tissue microarrays to measure the accumulation of neutrophils in the human AD brain using a range of markers including three antibodies against MPO, as well as antibodies against CD66B, and S100A8. For all of these markers, small spherical cells were observed, with little apparently glial or neuronal immunoreactivity, indicating that, as expected, they were detecting neutrophils (Fig. 1a-e). Additionally, cells stained for these antibodies had polymorphic nuclei, indicating that the cells they stained were indeed neutrophils (Additional file 1: Fig. S1). We found that there was a consistent increase in the abundance of these neutrophils within AD brains, as measured by all MPO antibodies, as well as antibodies against S100A8 and CD66B (Fig. 1a-e). Although other studies have detected S100A8 in other cell types [41], in our hands it was a reliable marker for neutrophils. In order to stain murine AD model brains, we tested a range of antibodies but found that only AF3667 gave specific signal in positive control stroke tissue (Additional file 1: Fig. S1). Analysis of neutrophil density by sex, age, and post-mortem delay revealed neutrophil accumulation was independent of these covariates, although we observed somewhat greater variability in males (Additional file 1: Fig. S2). We immunolabelled MPO and *S100A8* in the APP/PS1 mouse model, which has previously been shown to have cerebral neutrophil accumulation, and we observed an increase in neutrophil abundance in the cortex of both 4 and 12-month-old mice, although this was less striking than in human specimens (Fig. 1f, g). Regional analysis of mouse brains indicated that this effect was strongest in the cortex and hippocampus, although similar trends were observed in brainstem and cerebellum (Additional file 1: Fig. S3). Cortical and hippocampal densities were similar, and neutrophil accumulation in these regions was therefore quantified together. Finally, we observed an increase in the abundance of neutrophil markers calprotectin, of which S100A8 is a subunit, and MPO in AD brains by ELISA (Fig. 1h-j).

### MPO accumulation in the AD brain is driven by an increase in vascular neutrophils

**Fig. 2:**
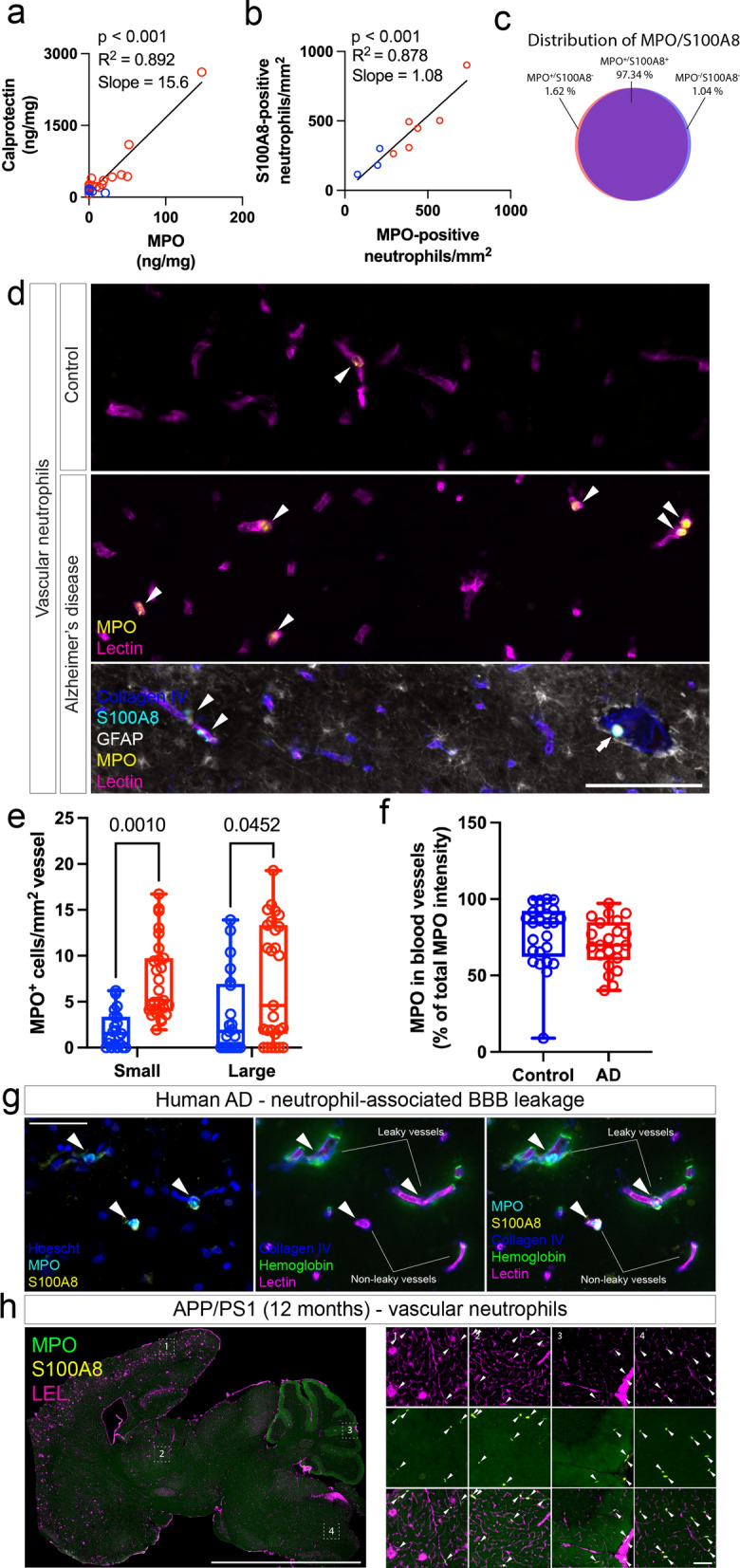
MPO accumulation in Alzheimer’s disease and APP/PS1 mice is driven by neutrophil accumulation. **a** ELISA was performed in neurologically normal and Alzheimer’s disease brains for the myeloperoxidase and calprotectin (S100A8/9 heterodimer). N = 7–13. P-values represent results of a Pearson’s correlation test. **b** FFPE-embedded human brain sections from the middle temporal gyrus (N = 3–6 per condition) were immunostained for MPO and S100A8. Correlation between MPO and S100A8 positive cells in independent sections. Values represent results of a Pearson’s correlation test. **c** Image analysis was used to identify cells as S100A8-positive, MPO-positive, or co-labelled. Venn diagram of overlap between S100A8 and MPO. Human brain tissue microarrays from the middle temporal gyrus (N = 21–28 per condition) were labelled with the vascular marker lectin and MPO. **d** Representative images and **e** quantification of the localisation of MPO within large (> 12 μm diameter) and small (< 12 μm diameter) lectin-positive vessels. Scale bar = 100 μm. Values represent results of a two-way ANOVA. **f** Quantification of the percentage of total MPO load present in blood vessels in AD and control brains. Wild-type or APP/PS1 mouse brains at 4 and 12 months of age were labelled for vascular marker tomato lectin (LEL) and neutrophil markers (MPO/S100A8). **g** Representative images of neutrophils associated with BBB leakage product hemoglobin in AD brains. Scale = 100 μm. **h** Representative images of MPO and S100A8-positive neutrophils in 12 month-old APP/PS1 vasculature. Scale bar = 5 mm, inset = 100 μm

Because we mainly observed neutrophil-like MPO staining in brain tissue, we wanted to determine if neutrophil abundance was the major factor driving the increase in MPO found in AD [18–20]. We therefore used alternative neutrophil markers calprotectin and S100A8 in immunostaining and ELISA, and found that these were positively correlated with MPO abundance (Fig. 2a, b). Indeed, in immunolabelling experiments at least 97% of MPO-positive cells were co-labelled with S100A8 (Fig. 2c). Previous reports have suggested that neutrophil accumulation in mouse AD models occurs primarily in the vasculature [12], and we therefore wished to determine the localisation of neutrophils relative to blood vessels. We used tissue microarrays to measure MPO localisation within the human AD brain and found that neutrophils were present in higher numbers in the human AD vasculature (Fig. 2d, e). Interestingly, when we compared the localisation of neutrophils by vessel segment, we observed that there were increases in both large and small vessels, however the magnitude of increase was greater in small vessels (< 12 μm diameter; Fig. 2d, e). In both control and AD brains we found that the majority of MPO labelling was contained in blood vessels (Fig. 2f). Interestingly, we also observed the presence of the BBB leakage product hemoglobin in close proximity to neutrophils found within blood vessels (Fig. 2g). In mouse AD model brains, we found a similarly striking vascular localisation of neutrophils in APP/PS1 mouse brains (Fig. 2h), as has been described previously [12].

**Fig. 3:**
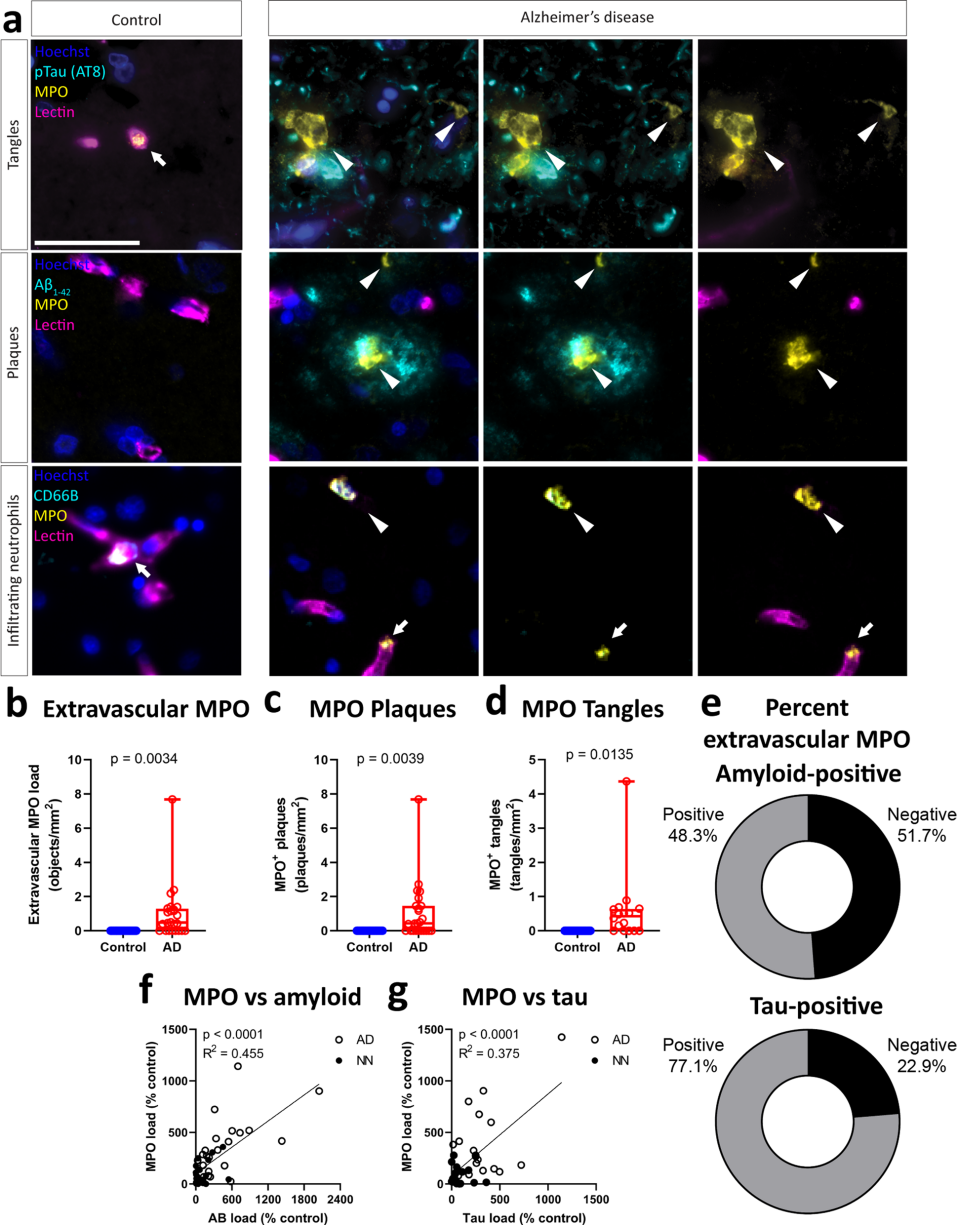
Non-vascular localisation of MPO in human Alzheimer’s disease. Tissue microarrays of human middle temporal gyrus were stained for MPO with pTau, amyloid-β, or CD66B. **a** Representative images of non-vascular MPO localisation associated with pTau tangles, amyloid-β plaques, and infiltrating CD66B-positive neutrophils. Scale = 25 μm. Quantification of the amount of **b** extravascular MPO, **c** MPO-positive plaques, and **d** MPO-positive tangles. Values represent results of an two-tailed Student’s T-test. **e** Percentage of extravascular MPO associated with plaques and tangles. Correlation of total MPO load in tissue with the load of **f** plaques and **g** tangles. Values represent results of a Pearson’s correlation test

Although the majority of neutrophils were found in the vasculature, we did observe rare extravascular CD66B-positive neutrophils indicating that infiltration does occur, albeit at a low rate (Fig. 3a, b). Furthermore, we observed sporadic deposition of MPO localised to amyloid plaques and tau tangles (Fig. 3a-d). Indeed, approximately half of the extravascular MPO-positive structures were localised to plaques, and a quarter to tangles (Fig. 3a, e). We observed significant correlations between both amyloid and tau deposition and MPO accumulation, although this was stronger for amyloid than tau (Fig. 3f, g).

### NETosis occurs in the AD vasculature

**Fig. 4:**
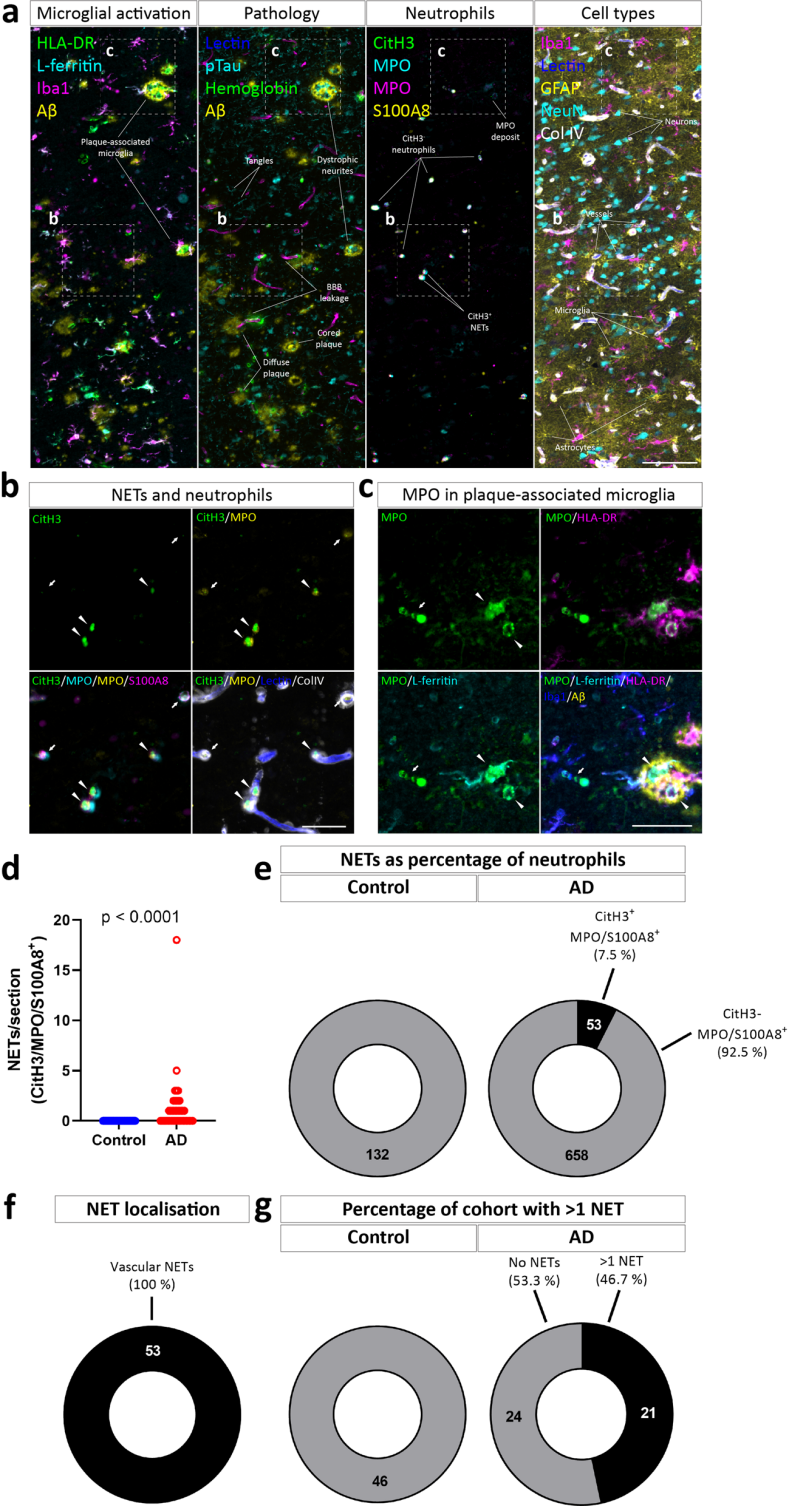
Identification of NETs within blood vessels in human Alzheimer’s disease. AD issue microarrays from the middle temporal gyrus (N = 45–46 per condition) were repeatedly immunostained, imaged, and stripped to build up a multiplexed panel of antibody labelling on the same section. This panel included the specific marker for NETs CitH3, as well as neutrophil/NET markers MPO and S100A8. **a** Representative images of all stains and insets of **b** NETs and **c** MPO-positive microglia. Scale bar = 250 μm, inset = 100 μm. **d** Quantification of the number of CitH3/MPO/S100A8-positive NETs in AD and control cores. P-values represent results of an two-tailed Student’s T-test. The proportion of **e** MPO/S100A8-positive neutrophils positive for CitH3, **f** localised in blood vessels, and **g** cases positive for NETs in AD and control

The formation of NETs is an important neutrophil effector function, and has been linked to vascular remodelling in the retina as well as stroke and traumatic brain injury [42–44]. We initially hypothesised that NETs would be present in the AD vasculature and MPO deposits around plaques in the brain. We used massively multiplexed immunolabelling of brains to stain a panel of pathological (Aβ, tau, NeuN), inflammatory (Iba1, L-ferritin, HLA-DR, GFAP), vascular (Lectin, Collagen IV, hemoglobin), and neutrophil markers (MPO, S100A8), alongside the specific NET marker CitH3 to categorically define NETs (Fig. 4a). We confirmed the specificity of the CitH3 antibody in brain tissue, detecting NETs in a murine model of stroke (Additional file 1: Fig. S1). We observed the presence of CitH3 exclusively localised to neutrophil markers, however only a sub-population of neutrophils was detected, indicating that it was indeed labelling NETs (Fig. 4b). We hypothesised that, analogous to their response to other large aggregated structures such as gallstones and cholesterol crystals, neutrophils may respond to aggregated forms of Aβ_1-42_ through NET formation [29, 30]. However, we did not detect NETosis in response to Aβ_1-42_ in any aggregation state in vitro (Additional file 1: Fig. S4). Furthermore, we only observed CitH3 localised to vascular neutrophils (Fig. 4b, f), but not plaque associated MPO-deposits as we originally hypothesised. MPO in these regions was colocalised with activated microglial markers Iba1, L-ferritin, and HLA-DR (Fig. 4c). Although NETs were present at higher levels in AD, they were only present in low abundance and in less than half of the cohort (Fig. 4d-g).

### Reduced endothelial glycocalyx in AD vessels

**Fig. 5:**
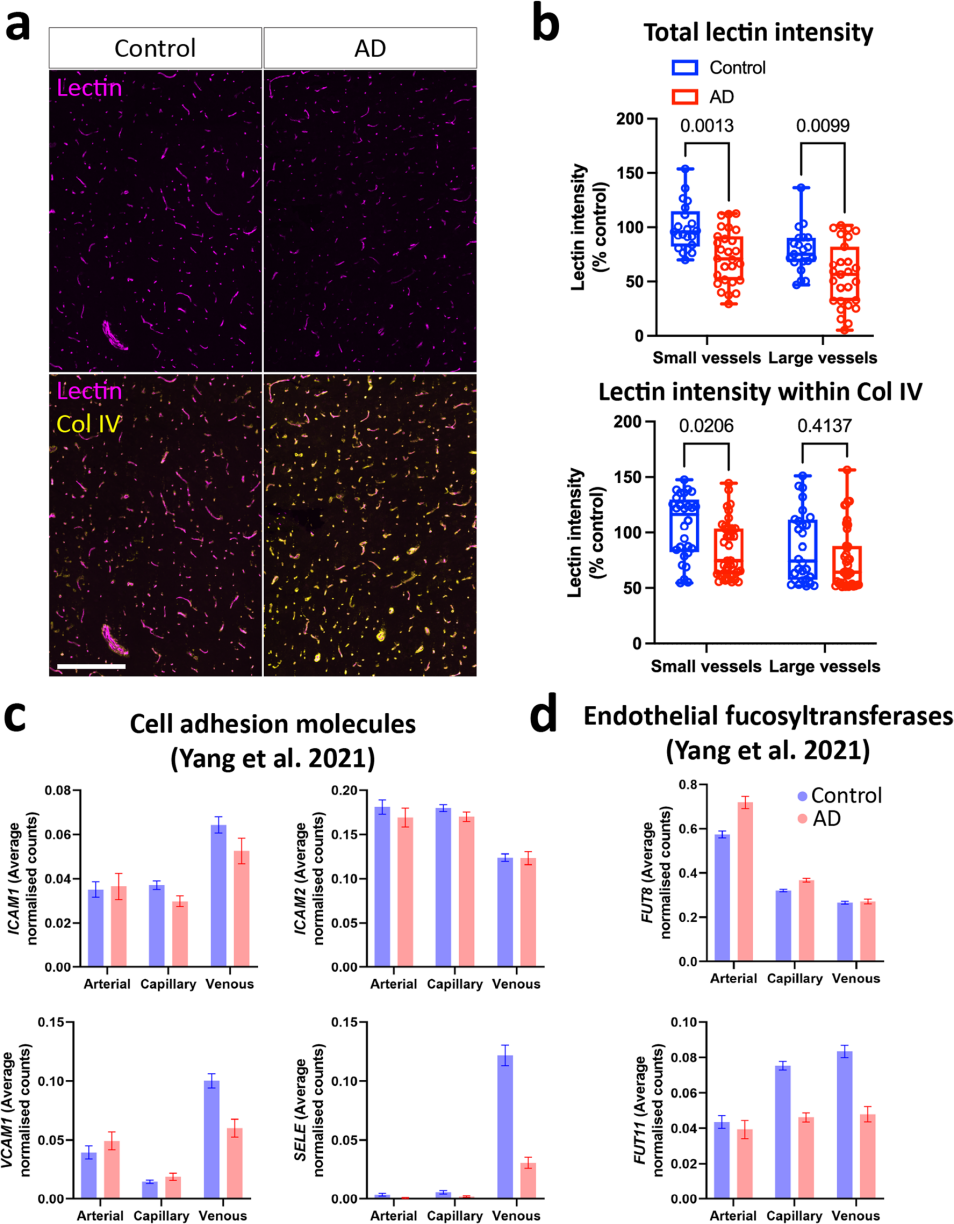
Reduction in endothelial glycocalyx in AD brain. AD tissue microarrays from the middle temporal gyrus (N = 21–37 per condition) were labelled for the total vessel marker collagen IV, and endothelial glycocalyx marker UEA1 lectin. **a** Representative images of lectin immunolabelling, and **b** quantification of lectin intensity in blood vessels in control and AD brains, segregated by vessel size. Scale = 250 μm. P-values represent results of an two-way ANOVA. Single nuclear RNA-seq from the human AD vasculature were obtained from https://twc-stanford.shinyapps.io/human_bbb/, and genes involved in **c** cell adhesion processes, and **d** fucosylation of the endothelial glycocalyx evaluated

During previous experiments (Figs. 2, 3, 4), we observed a reduction in the intensity of staining for UEA1 lectin, a marker for fucosylation in the glycocalyx, in human AD vessels. Indeed, image analysis indicated that there was significantly reduced UEA1 staining intensity in AD, irrespective of vessel size (Fig. 5a, b). To ensure results were not confounded by changes to vascular density, we used collagen IV as a total vascular marker, and measured lectin intensity within collagen-positive structures, again segregating large and small vessels. There was a similar reduction in the lectin staining, particularly in small vessels, however a similar trend was observed in large vessels too (Fig. 5a, b). We also interrogated potential mechanisms through which attachment may be mediated using a single-cell RNAseq atlas of the AD vasculature [40]. Interestingly, although there were no increases in the expression of cell adhesion molecules (*ICAM1, ICAM2, VCAM1, SELE*) in endothelial cells, there was a decrease in *FUT11*, an enzyme associated with the production of the glycocalyx (Fig. 5c, d) [40].

## Discussion

While increased MPO [11, 18–20] and cerebral neutrophil accumulation [10–13] are well-established features of AD, there have been several reports of unusual cellular sources and localisations of MPO. Previous reports have primarily focussed on the accumulation of neutrophils in mouse models [10–13], or have used a limited panel of antibodies [11, 18, 19]. We used tissue from a large human cohort and mouse tissue to show that MPO deposition in the AD brain is primarily driven by neutrophil accumulation. The majority of this MPO is contained in vascular-associated neutrophils, confirming reports from animal models of AD [12]. Furthermore, we find evidence for vascular NETosis in human AD. Finally, we observe a decrease in staining for the glycocalyx using UEA-1 lectin to indicate that reductions in the endothelial glycocalyx may drive neutrophil accumulation in the AD vasculature.

Several reports have indicated that MPO may be produced by non-neutrophil cells, including microglia [19], astrocytes [20], and neurons [19]. Using multiple different antibodies we confirmed that the majority of MPO staining was confined to neutrophils. It should be noted that, although S100A8 can be expressed in other cell types, MPO was also found almost exclusively colocalized with other neutrophil markers including CD66B. Furthermore the nuclei associated with MPO were polymorphonuclear. We did, however, observe low levels of MPO in plaque-associated microglia, reinforcing reports that it may be a microglial activation marker [46]. Since the bulk of MPO labelling was confined to neutrophils, we concluded that the increase of MPO in AD is mainly driven by the accumulation of neutrophils in the brain. Strikingly, we predominantly observed neutrophils present within lectin-positive blood vessels, and only very rarely in the parenchyma in both a mouse model of amyloidosis and in human AD.

Our work corroborates findings from mouse AD models in human AD, suggesting that neutrophil plugging of capillaries leads to blood flow reductions in AD [12, 13]. Importantly, neutrophil plugging of the vasculature occurred early in this mouse model, and the removal of neutrophil plugs led to improved cerebral blood flow and cognition [12, 13]. While our work was performed on tissue from end-stage disease, this suggests that neutrophil plugging may also be present early in human AD. The enhanced neutrophil-vascular interactions observed in AD suggest that either neutrophils or the vasculature become ‘stickier’. It is also important to note that human samples are processed differently to those in mouse studies, with a significant delay between death and perfusion-fixation, and this may influence the numbers of neutrophils present in the vasculature. Despite this, tissue processing is consistent between human samples, and so results remain internally consistent.

There have been conflicting reports, some suggesting that the cell adhesion molecules ICAM1 and VCAM1 are enhanced in the AD vasculature [11, 47], but more recent findings indicate that expression of genes involved in neutrophil attachment are unchanged in human AD endothelia [40]. Interestingly, although vascular neutrophils were increased in APP/PS1 mice, the degree of attachment was lower than in AD, suggesting that although vascular changes are present, they are not equivalent [40]. Furthermore, we find that UEA-1 lectin staining of the endothelial glycocalyx is decreased in AD. Indeed, loss of endothelial glycocalyx in a mouse model of subcortical vascular dementia was responsible for capillary stalling [48]. Furthermore, hyaluronan, a marker of glycocalyx damage, was elevated in the cerebrospinal fluid of AD and vascular dementia patients [49, 50]. Circulating neutrophils have been reported to be more activated in AD [51], and they can also alter the endothelial glycocalyx [52, 53], so it is possible that these changes to neutrophil activation state are responsible for glycocalyx changes and vascular attachment. However, we speculate that glycocalyx thinning may result from reduced production with reduced expression of *FUT11*, a gene involved in glycocalyx production in AD endothelial cells [40]. This may allow for enhanced neutrophil attachment, even in the absence of strong cell adhesion molecule expression. Furthermore, in the absence of strong cell adhesion molecule expression, attachment would not facilitate neutrophil infiltration, and may explain why neutrophils remain in the vasculature. In human samples though, it is worth noting that we could not distinguish the means through which neutrophils had become present in vessels. Indeed, it is possible that the observed accumulation of neutrophils here may have been the result of them becoming trapped, rather than adhering the vessel wall. Importantly, although human and mouse brains were processed differently, we did observe similar enhancement of vascular associated neutrophils in AD and the APP/PS1 mouse model of AD indicating that our observations are unlikely to be the result of a post-mortem artefact. It will be important to further validate changes to the AD glycocalyx composition, and the expression of the enzymes that synthesise it. Indeed, it may be possible to modify endothelial glycocalyx synthesis to prevent neutrophil adhesion to capillaries in AD.

The accumulation of neutrophils and MPO in the brain vasculature has important consequences for the surrounding cells [22]. Indeed, cells at the borders of the brain can have profound effects on cognition and pathology. MPO binds strongly to the vascular endothelium through its interactions with the negatively charged glycocalyx in other chronic inflammatory diseases, including heart disease, to cause endothelial dysfunction [52, 54, 55]. Here, too, we observe BBB dysfunction at sites where neutrophils are attached to blood vessels. Furthermore, MPO impairs the barrier function of brain endothelial cells through the production of HOCl [56]. HOCl is highly reactive and consumed close to its site of production [23]. It is possible that longer-lived secondary oxidants such as chloramines mediate the effects of HOCl on the vasculature [23]. In the presence of thiocyanate concentrations present in plasma, MPO also generates HOSCN [57]. We have found that this longer lived, thiol-selective oxidant can impact endothelial cells at sub-lethal doses [58], including disruption of BBB function [59]. In the context of AD, it is possible that neutrophil-vascular interactions are an important mechanism through which BBB dysfunction is established [22].

We observed very little parenchymal MPO indicating that the majority of neutrophil attachment in AD does not lead to neutrophil infiltration. However, it is clear that there is a limited degree of neutrophil infiltration into the AD brain, supporting previous reports [10, 11]. It is interesting to note that we did not observe profound infiltration of neutrophils into the brains of APP/PS1 mice. While this contradicts results in tauopathy and 5XFAD mice, it does align with other reports from the APP/PS1 model, indicating that strain may influence the infiltration of neutrophils in AD models [12]. Furthermore, we detected parenchymal MPO deposits associated with AD pathology. These MPO deposits were larger and morphologically different to staining patterns observed in neutrophils. When activated, neutrophils are capable of forming NETs, creating a DNA–protein matrix that has powerful immunostimulatory properties [28]. We initially hypothesised that these MPO deposits were NETs however we could not observe colocalisation of other neutrophil or NET markers. Plaque-associated MPO deposits were colocalised with markers of activated microglia (HLA-DR, L-ferritin), albeit at much lower levels than neutrophils. Indeed, MPO may be an activation marker in microglia in its own right [46]. It is also possible that parenchymal NETs cannot be detected through CitH3 labelling, as the citrullination occurs in a region of the histone tail that is cleaved during NETosis and is a more robust marker of early, rather than mature, NETs [60]. Antibodies to NET-specific modifications in mature NETs could be used to detect this phenomenon [60].

We observed NETosis in the AD vasculature, although this was not ubiquitous and the presence of NETs was rarer than in stroke, our positive control. There have been other reports of NETosis in sterile neurological diseases, including stroke and traumatic brain injury, where they are involved in vascular remodelling [42, 43]. While there are preliminary reports of NETosis in the AD brain [11], their localisation has not been interrogated. Recent reports indicate that senescent endothelial cells attract and promote NETosis to trigger vascular remodelling in the retina following oxygen-induced retinopathy [44], and that NETs also play a role in vascular remodelling following stroke [43]. It is possible that a similar phenomenon occurs in AD, leading to vascular regression [40, 61]. Recent reports suggest that CNS-associated neutrophils are derived from multiple sources, including the blood and adjacent skull bone marrow [62, 63]. The vascular association of neutrophils observed here suggests their origin from the circulation, which may be associated with activated neutrophil phenotypes in AD that are more prone to NETosis [62].

The localisation of neutrophils and MPO within cerebral vessels suggests that they may be a peripheral target with the potential to enhance cognitive function in AD. Indeed, preventing leukocyte tethering to the brain vasculature improves cognition in aging [64], as well as in models of AD [11–13]. Furthermore, the presence of NETs, and therefore extracellular MPO at the brain vasculature represents a novel AD-associated process. It may be possible to prevent MPO release by targeting NETosis, degrading NETs, or by addition of molecules that sequester MPO from the glycocalyx to ‘wash’ it off the endothelium without modulating potentially beneficial functions within the neutrophil cytoplasm [57,66]. Although the majority of neutrophils in AD are present at a brain barrier and not within the brain itself, they may still have important effects on cognition [9, 11, 12], so targeting inflammation in BBB endothelial cells or through modulation of neutrophils may be a useful therapeutic target in AD [15].

## Conclusions

**Fig. 6:**
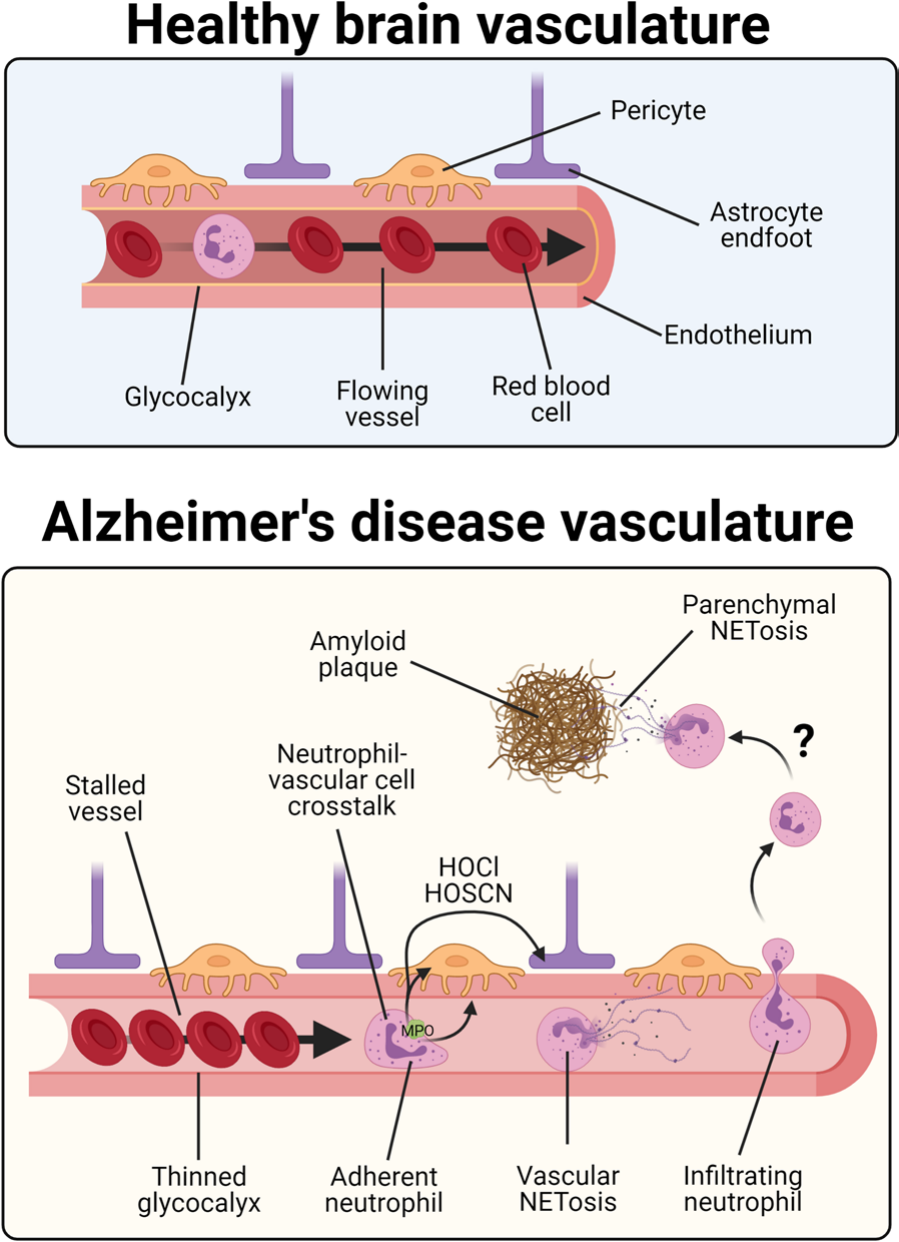
Effects of neutrophil-vascular interations in AD. In healthy brains, neutrophils flow freely through vessels with limited interactions with endothelial cells enabling adequate perfusion. During Alzheimer's disease, reductions in the glycocalyx may enhance the non-productive attachment of neutrophils to endothelial cells. These attached neutrophils can cause the stalling of vessels, undergo NETosis, and may lead to oxidative stress and blood-brain barrier breakdown. Reducing neutrophil-vascular interactions may be beneficial to improve vascular function in AD

Here, we confirm data from animal models in the human AD brain suggesting that neutrophils accumulate, and are associated with blood vessels in AD where they may contribute to vascular stalling and BBB leakage (Fig. 6). We also show that neutrophils and NETs are the key source of MPO in the brain during AD which may represent an important mechanism through which BBB inflammation influences oxidative stress in AD.

## Supplementary Information

**Additional file 1**. **Figure S1**: MPO labelling is predominantly confined to neutrophils.** Figure S2**: Neutrophil accumulation is independent of sex, age, and post-mortem delay.** Figure S3**: Regional analysis of neutrophil accumulation in APP/PS1 mice.** Figure S4**: Amyloid-β1-42 does not induce NETosis in vitro.** Figure S5**: Specificity of antibody labelling across multiple rounds of multiplexed immunohistochemistry.** Table S1**: Case details of tissue used in tissue microarray and immunohistochemistry experiments.** Table S2**: Details of antibodies used for these studies.

## Data Availability

The data presented in this study are included in the manuscript and supplementary material. Additional data that are not included can be made available upon reasonable request to the corresponding author.

## Abbreviations

Aβ: Amyloid beta
AD: Alzheimer’s disease
APP/PS1: Amyloid precursor protein/presenilin-1 mouse AD model
BBB: Blood–brain barrier
CitH3: Citrullinated histone H3
ELISA: Enzyme-linked immunosorbent assay
FUT11: Fucosyltransferase 11
GFAP: Glial fibrillary acid protein
HLA-DR: Human Leukocyte Antigen–DR isotype
HOCl: Hypochlorous acid
HOSCN: Hypothiocyanous acid
Iba1: Allograft inflammatory factor 1
ICAM1: Intercellular cell adhesion molecule-1
NET: Neutrophil extracellular trap
S100A8: S100 calcium-binding protein A8
SELE: Selectin-endothelial
UEA-1: *Ulex europaeus* Agglutinin-1
VCAM1: Vascular cell adhesion molecule-1
