# Supplementary material for: Neutrophil-vascular interactions drive myeloperoxidase accumulation in the brain in Alzheimer’s disease
Source: Acta Neuropathol Commun. 2022 Mar 24;10:38. doi: 10.1186/s40478-022-01347-2 (PMC8944147; doi:10.1186/s40478-022-01347-2)
Supplement: Supplementary file 1 — Additional file 1. Figure S1: MPO labelling is predominantly confined to neutrophils. Figure S2: Neutrophil accumulation is independent of sex, age, and post-mortem delay. Figure S3: Regional analysis of neutrophil accumulation in APP/PS1 mice. Figure S4: Amyloid-β1-42 does not induce NETosis in vitro. Figure S5: Specificity of antibody labelling across multiple rounds of multiplexed immunohistochemistry. Table S1: Case details of tissue used in tissue microarray and immunohistochemistry experiments. Table S2: Details of antibodies used for these studies. [file 40478_2022_1347_MOESM1_ESM.pdf]

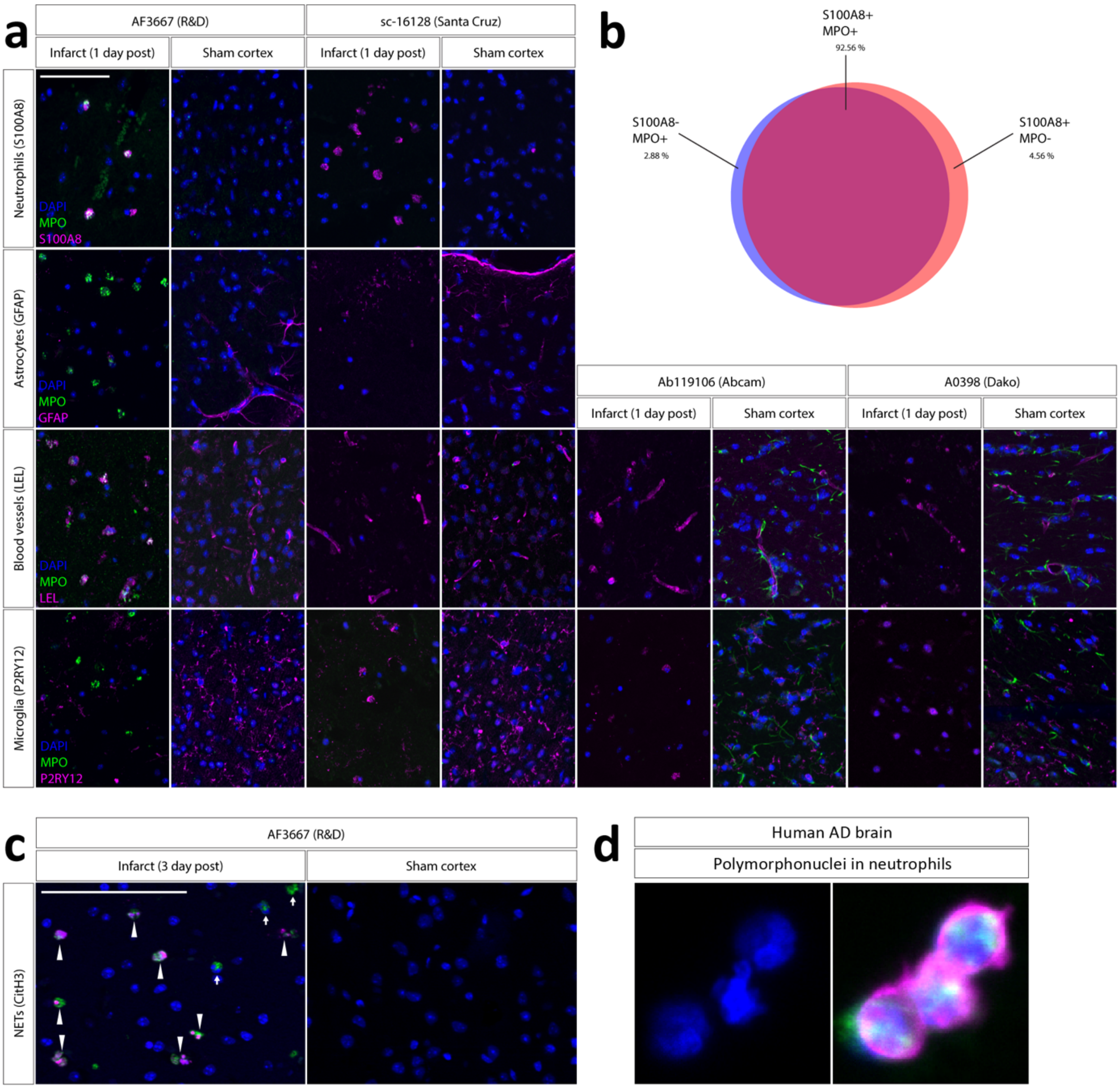

Figure S1: MPO labelling is predominantly confined to neutrophils. Stroke was induced in mice by photothrombosis, and FFPE-embedded brains were stained with four different antibodies against MPO, as well as other cellular markers. a) Representative images of MPO labelling with S100A8, GFAP, tomato lectin, and P2RY12. Scale = 100 µm. b) Colocalisation of MPO (AF3667) with S100A8. c) Positive control immunolabelling for CitH3/MPO-positive NETs in stroke tissue (3 days post-infarct). Scale = 100 µm. d) Polymorphic nuclei in S100A8, MPO positive neutrophils.

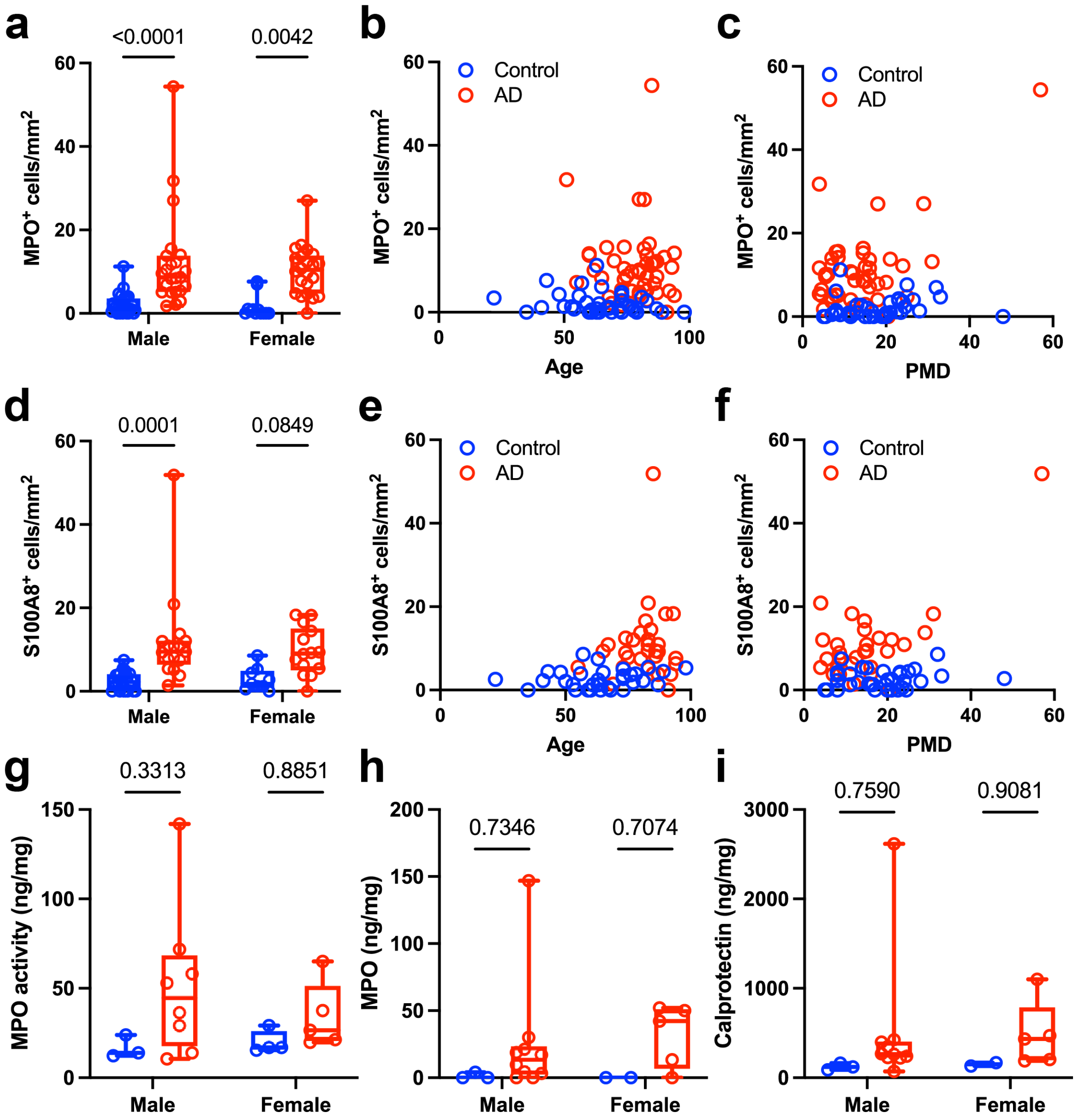

Figure S2: Neutrophil accumulation is independent of sex, age, and post-mortem delay. Neutrophil numbers in TMAs were quantified by both MPO and S100A8 staining. a) Neutrophil accumulation, measured by MPO staining, stratified by sex and disease state. Scatterplot of neutrophil accumulation, measured by MPO staining, as a function of b) age at death and c) post-mortem delay. P-values represent results of a two-way ANOVA. d) Neutrophil accumulation, measured by S100A8 staining, stratified by sex and disease state. Scatterplot of neutrophil accumulation, measured by S100A8 staining, as a function of e) age at death and f) post-mortem delay. P-values represent results of a two-way ANOVA. Neutrophil markers MPO and calprotectin were measured in the brain by ELISA. g-i) MPO activity, abundance, and calprotectin abundance in the brain stratified by sex. P-values represent results of a two-way ANOVA.

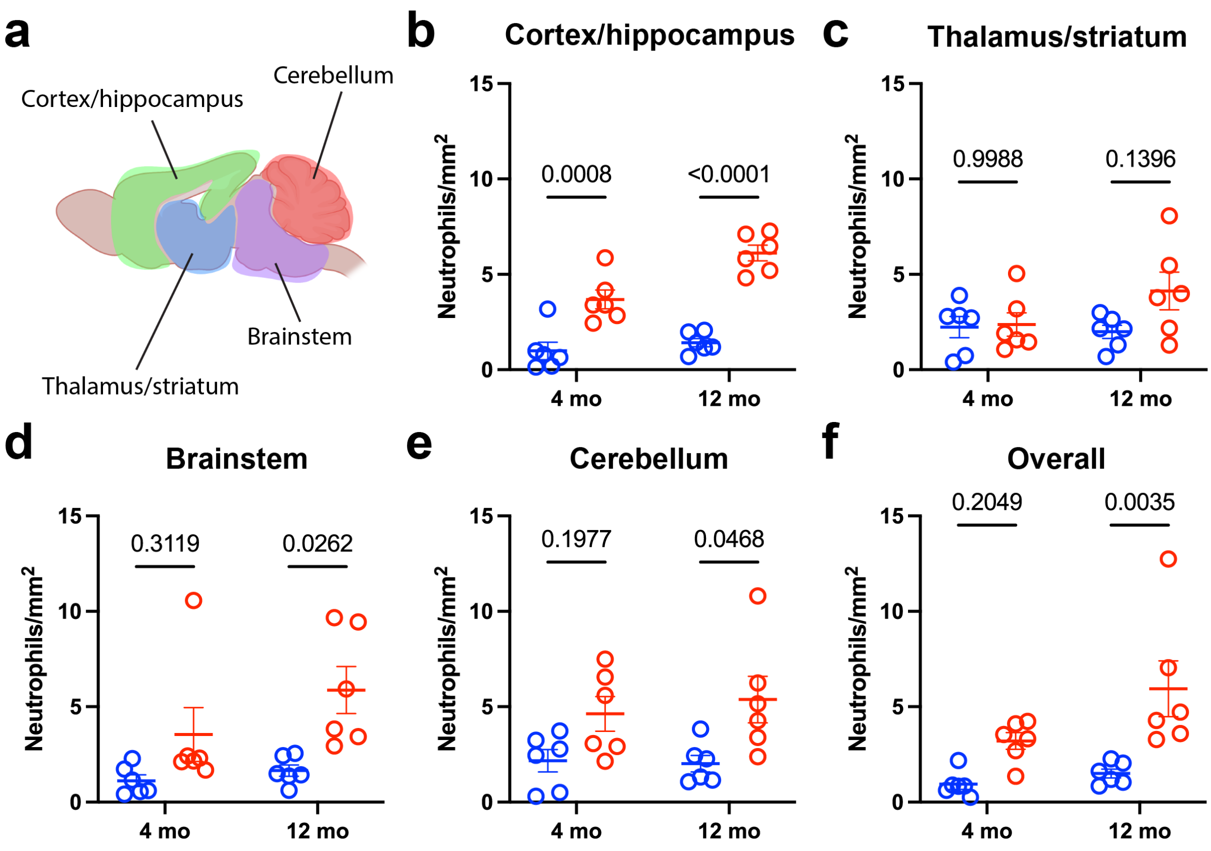

Figure S3: Regional analysis of neutrophil accumulation in APP/PS1 mice. Brains were immunostained and neutrophil accumulation quantified. a) Regions investigated in this study. Neutrophil density in the b) cortex and hippocampus, c) thalamus and striatum, d) brainstem, e) cerebellum, and f) overall.

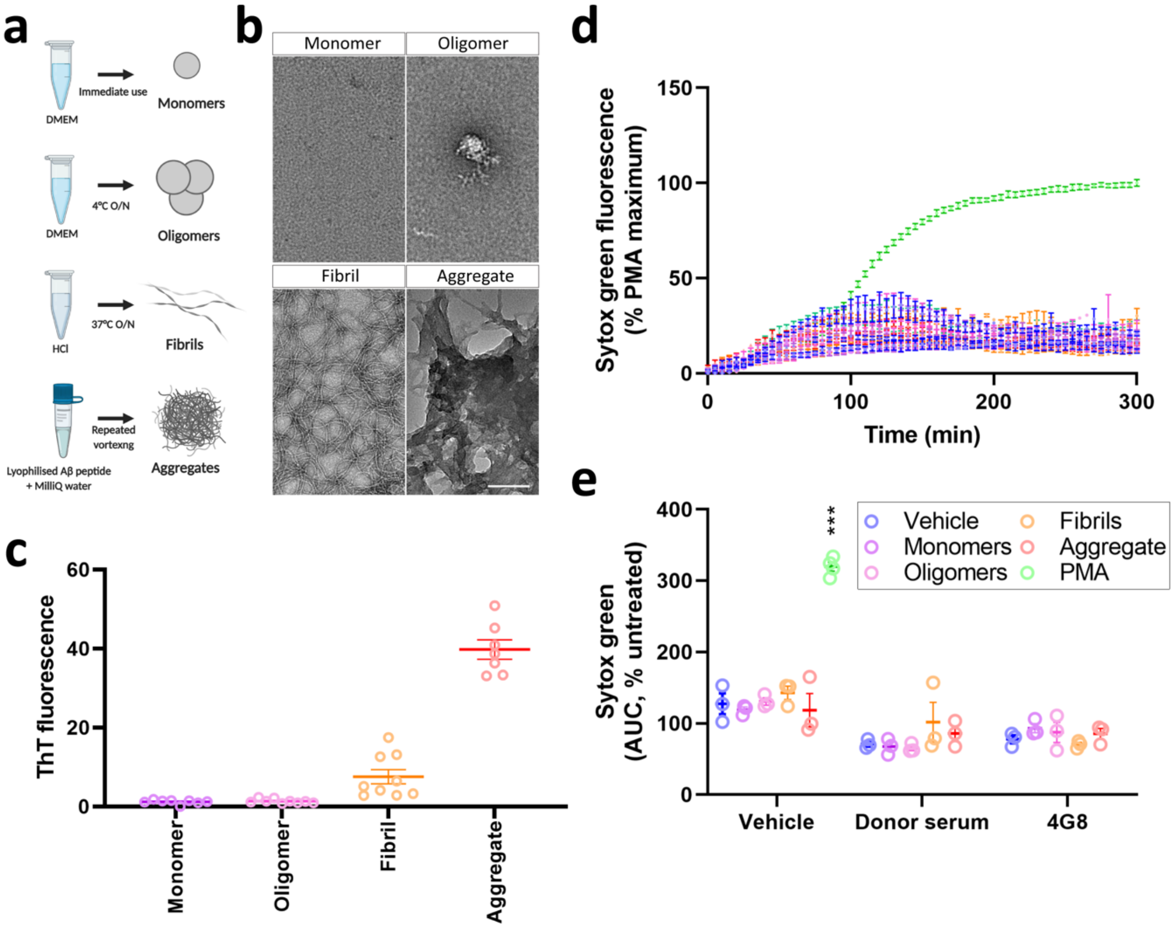

Figure S4: Amyloid-β_{1-42} does not induce NETosis in vitro. a) Aβ_{1-42} was differentially aggregated in vitro, and aggregation states analysed by b) electron microscopy and c) thioflavin T assay. Scale = 100 nm. Amyloid aggregation states were added to neutrophils in the presence of donor serum or anti-Aβ antibody 4G8, and the Sytox green NETosis assay performed with PMA (20 nM) as a positive control. d) Traces and e) area under the curve quantification of Sytox green changes in neutrophils treated with Aβ_{1-42}.

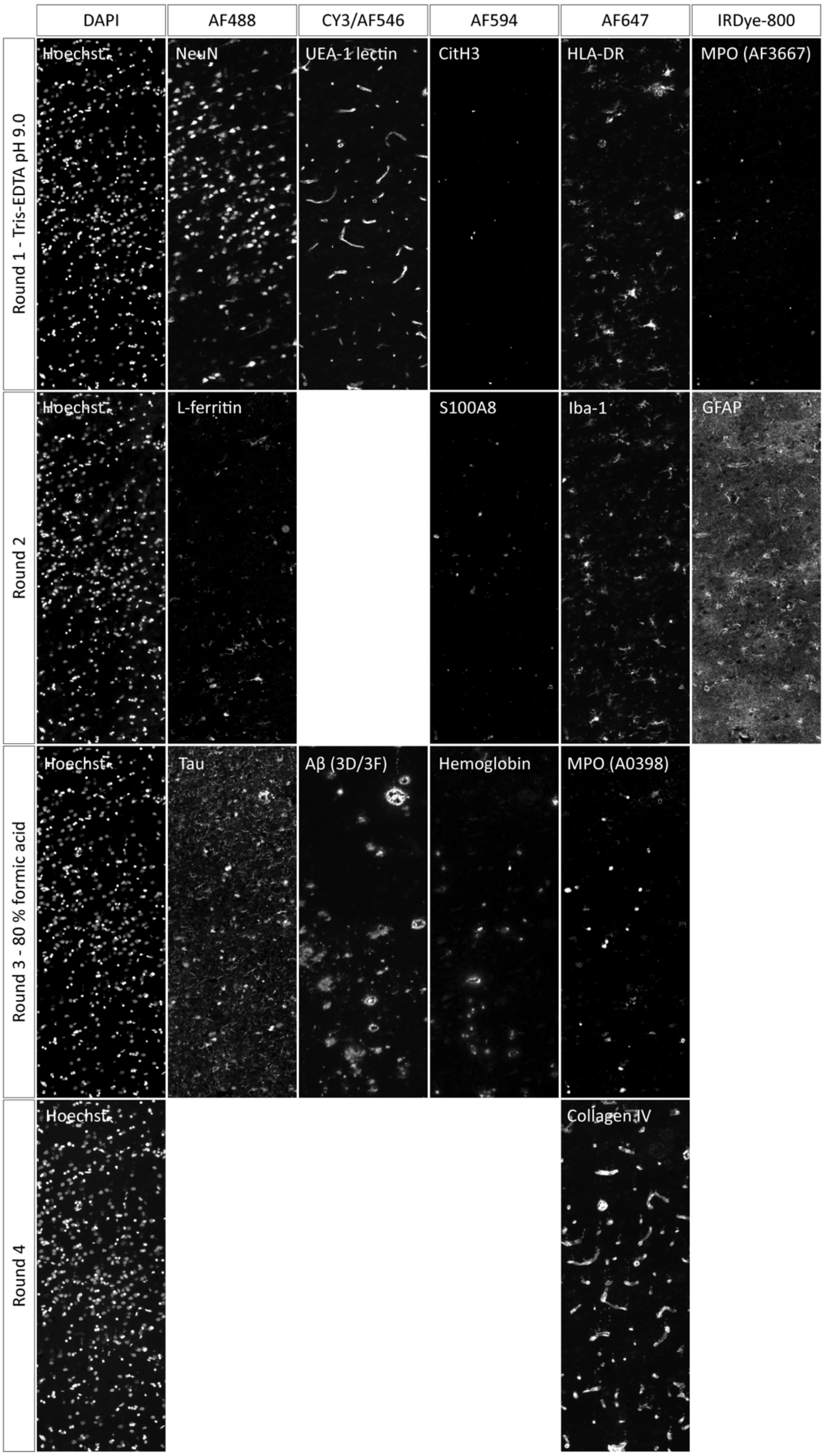

Figure S5: Specificity of antibody labelling across multiple rounds of multiplexed immunohistochemistry. Individual monochrome images used to create Figure 4a separated by channel and staining round.

Table S1: Case details of tissue used in tissue microarray and immunohistochemistry experiments. COD = cause of death, MTG = middle temporal gyrus, NN = neurologically normal, AD = Alzheimer's disease, PMD = post-mortem delay.

Case	Region	Age	Pathology	Sex	PMD (h)	Notes
**02F/393**	**MTG**	87	NN	F	11	
**4680**	**MTG**	80	NN	M	12	
**4734**	**MTG**	79	NN	M	8	
**6013**	**MTG**	69	NN	F	11.5	
**H121**	**MTG**	64	NN	F	5	
**H122**	**MTG**	72	NN	F	9	
**H123**	**MTG**	78	NN	M	13	
**H127**	**MTG**	59	NN	F	21	
**H136**	**MTG**	75	NN	M	13	
**H137**	**MTG**	77	NN	M	12	
**H139**	**MTG**	73	NN	M	5.5	
**H144**	**MTG**	76	NN	M	19	
**H145**	**MTG**	54	NN	M	8	
**H148**	**MTG**	64	NN	M	7	
**H150**	**MTG**	78	NN	M	11	
**H151**	**MTG**	64	NN	F	5	COD: coronary atherosclerosis. Non-specific diffuse beta-amyloid plaques = age related; No AD; No cortical or LB; CN & CB unremarkable.
**H152**	**MTG**	79	NN	M	18	
**H153**	**MTG**	76	NN	M	8	
**H155**	**MTG**	61	NN	M	7	
**H156**	**MTG**	89	NN	M	19	
**H159**	**MTG**	53	NN	M	16.5	COD: ischemic heart disease. Control specimen, normal for age.
**H160**	**MTG**	77	NN	M	23	
**H164**	**MTG**	73	NN	M	13	
**H165**	**MTG**	43	NN	F	26	COD: nitrogen poisoning. No significant histological abnormalities.
**H167**	**MTG**	51	NN	M	23.5	COD: asphyxia. Hepatitis C positive.
**H168**	**MTG**	63	NN	M	9	COD: ischemic heart disease. No significant histological abnormalities.
**H169**	**MTG**	81	NN	M	24	COD: asphyxia. No LBD; low AD change (A2 B0 C1); cerebral amyloid angiopathy; Tau+ grain-like path in CA1.
**H170**	**MTG**	60	NN	M	17	COD: ischemic heart disease. No significant histological abnormalities.
**H174**	**MTG**	59	NN	M	24.5	COD: aortic aneurysm. No significant histological changes.
**H177**	**MTG**	22	NN	M	21	COD: asphyxia. Control specimen: No significant pathological changes.
**H180**	**MTG**	73	NN	M	33	
**H183**	**MTG**	61	NN	M	13	COD: multiple organ failure. Control specimen: No significant pathological changes.
**H184**	**MTG**	35	NN	M	20	COD: electrocution. Control specimen: No significant pathological changes.
**H185**	**MTG**	50	NN	M	28	COD: obscure causes. Control specimen: No significant pathological changes.
**H186**	**MTG**	68	NN	M	21	COD: ischemic heart disease. Control specimen: No significant pathological changes.
**H187**	**MTG**	98	NN	F	15	
**H188**	**MTG**	83	NN	M	17	
**H190**	**MTG**	72	NN	F	19	
**H191**	**MTG**	77	NN	M	20	

**H192**	**MTG**	65	NN	F	23	COD: ischemic heart disease. No significant histological changes, Hepatitis B44 positive.
**H194**	**MTG**	68	NN	M	22.5	COD: coronary atherosclerosis. No significant histological abnormalities.
**H195**	**MTG**	65	NN	M	18	COD: ischemic heart disease. No significant histological abnormalities.
**H196**	**MTG**	85	NN	F	15	
**H198**	**MTG**	67	NN	F	27	
**H200**	**MTG**	56	NN	M	23	COD: asphyxia. No significant histological abnormalities.
**H202**	**MTG**	83	NN	M	14	
**H209**	**MTG**	48	NN	M	23	COD: ischemic heart disease. Appearances unremarkable for age.
**H215**	**MTG**	67	NN	F	23.5	COD: ischemic heart disease. Control specimen: no significant histological abnormalities.
**H226**	**MTG**	73	NN	F	48	COD: mesothelioma. Cerebral age-related change.
**H230**	**MTG**	57	NN	F	32	COD: carcinomatosis (renal). Age-related cerebral changes.
**H231**	**MTG**	65	NN	M	8	COD: ischemic heart disease. No significant histological abnormalities.
**H238**	**MTG**	63	NN	F	16	COD: dissecting aortic aneurysm. No significant histological abnormalities.
**H239**	**MTG**	64	NN	M	15.5	COD: ischemic heart disease. No significant histological abnormalities.
**H240**	**MTG**	73	NN	M	26.5	COD: ruptured aneurysm, abdominal haemorrhage. Mild cerebral cortical abnormalities. Sparse neurofibrillary tangles.
**H242**	**MTG**	61	NN	M	19.5	COD: coronary atherosclerosis. Relatively unremarkable; Diffuse beta amyloid plaques in MTG, cerebral amyloid angiopathy.
**H245**	**MTG**	63	NN	M	20	COD: asphyxia. No significant abnormality identified.
**Avg**		67.4		28.6	17.5	
**AZ33**	**MTG**	65	AD	M	20	COD: hypostatic pneumonia. CERAD: Definite Alzheimer's disease. Atrophy: mid-1, Tangles: mild-1, Plaques: mod-2, ARP: C.
**AZ34**	**MTG**	74	AD	F	18	ARP: C.
**AZ37**	**MTG**	83	AD	M	4	ARP: B.
**AZ38**	**MTG**	80	AD	M	5.5	ARP: C.
**AZ39**	**MTG**	74	AD	M	12	ARP: C.
**AZ42**	**MTG**	60	AD	M	7	ARP: C.
**AZ43**	**MTG**	80	AD	M	21	ARP: B.
**AZ45**	**MTG**	82	AD	M	4.5	ARP: B.
**AZ46**	**MTG**	82	AD	F	22	ARP: B.
**AZ52**	**MTG**	68	AD	F	36	ARP: C.
**AZ55**	**MTG**	51	AD	M	4	ARP: B.
**AZ57**	**MTG**	82	AD	F	14.5	ARP: A.
**AZ58**	**MTG**	75	AD	M	20	ARP: C.
**AZ59**	**MTG**	83	AD	M	15	ARP: A.
**AZ61**	**MTG**	87	AD	F	7.5	ARP: C.
**AZ64**	**MTG**	67	AD	M	8	ARP: C.
**AZ65**	**MTG**	77	AD	F	16	ARP: C.
**AZ68**	**MTG**	68	AD	F	7	ARP: C.
**AZ71**	**MTG**	62	AD	F	6	ARP: C.
**AZ72**	**MTG**	70	AD	F	7	Braak: V, ARP: C.

**AZ73***	**MTG**	87	AD	F	14.5	Mixed Pathology: Alzheimer's disease & Cortical Lewy Body Disease Braak: IV, Atrophy: 1/3, Tangles: 2/3, Plaques: 2/3, ARP: B
**AZ74**	**MTG**	85	AD	F	16	Braak: VI, ARP: C.
**AZ75**	**MTG**	85	AD	M	25	Braak: V/VI, ARP: C.
**AZ77**	**MTG**	81	AD	F	16	Braak: IV/VI, ARP: B.
**AZ78**	**MTG**	87	AD	F	7	COD: general inanition, dementia. CERAD: probable Alzheimer's disease. Braak: 3/6, Atrophy: 2/3, Tangles: 2/3, Plaques: 1/3, ARP: B.
**AZ80**	**MTG**	77	AD	M	4.5	Braak: IV/IV, ARP: C.
**AZ81**	**MTG**	82	AD	F	18	Braak: IV/IV, ARP: C.
**AZ82**	**MTG**	80	AD	F	18	Braak: IV/IV, ARP: C.
**AZ83**	**MTG**	60	AD	F	16	Braak: IV/IV, ARP: C.
**AZ85**	**MTG**	85	AD	M	57	COD: AD, prostate cancer. CERAD: probable Alzheimer's disease. Braak: 4/6, Atrophy: 0/3, Tangles: 2/3, Plaques: 1/3, ARP: B.
**AZ86**	**MTG**	92	AD	M	8.5	COD: bronchopneumonia, chronic renal failure. CERAD: possible Alzheimer's disease. Braak: 3/6, Atrophy: 0/3, Tangles: 1/3, Plaques: 1/3, ARP: A.
**AZ87**	**MTG**	73	AD	M	5	
**AZ88**	**MTG**	83	AD	M	21	COD: pneumonia. CERAD: Definite Alzheimer's disease. Braak: 4/6; Atrophy: 2/3, Tangles: 3/3, Plaques: 3/3, ARP: C.
**AZ89**	**MTG**	80	AD	F	25	COD: advanced dementia. CERAD: Definite Alzheimer's disease. Braak: 6/6; Atrophy: 3/3, Tangles: 3/3, Plaques: 3/3, ARP: C.
**AZ90**	**MTG**	73	AD	M	4	COD: gastrointestinal haemorrhage. CERAD: Definite Alzheimer's disease. Braak: 4/6; Atrophy: 3/3, Tangles: 3/3, Plaques: 3/3, ARP: C.
**AZ91**	**MTG**	80	AD	M	29	COD: sepsis, aspiration pneumonia. CERAD: Definite Alzheimer's disease. Braak: 5/6; Atrophy: 2/3, Tangles: 3/3, Plaques: 3/3, ARP: C.
**AZ92**	**MTG**	93	AD	F	11.5	COD: bronchopneumonia. CERAD: Probable Alzheimer's disease. Braak: 4/6, Atrophy: 3/3, Tangles: 3/3, Plaques: 3/3, ARP: B.
**AZ93**	**MTG**	83	AD	M	15	COD: AD dementia. CERAD: Probable Alzeimer Disease. Mod plaque density; Braak Stage V.
**AZ95**	**MTG**	69	AD	M	12	COD: bronchopneumonia, aspiration pneumonia. CERAD: Alzheimer's disease. Braak: 5/6, Atrophy: 3/3, Tangles: 2/3, Plaques: 3/3, ARP: C.
**AZ96**	**MTG**	74	AD	F	8.5	COD: metastatic cancer, likely gastric. CERAD: Alzheimer's disease Braak: 5/6; Atrophy: 3/3, Tangles: 3/3, Plaques: 3/3, ARP: C.
**AZ98**	**MTG**	91	AD	F	20.5	COD: Alzheimer’s dementia, atrial fibrillation. Alzheimer's disease.
**AZ99**	**MTG**	94	AD	F	8.5	COD: multiple organ systems failure. Alzheimer’s-type neuropath. change (A3, B3, C2), Braak V-VI; small vessel cerebrovascular wth focal lacunar

						infarction; focal cerebral amyloid angiopathy.
**AZ101**	**MTG**	75	AD	M	12.5	COD: right lower lobe pneumonia. AD neuopathology. change (A3, B3, C2), Braak VI; cerebral amyloid angiopathy; old infarction in R temporo-occipital; small vessel cerebrovascular disease (CT).
**AZ102**	**MTG**	84	AD	F	14.5	COD: lower respiratory tract infection. Alzheimer's-type neuropathological change (A3, B2, C2), Braak IV; hyaline arteriosclerosis.
**AZ103**	**MTG**	87	AD	M	<24	COD: cerebrovascular event. Alz-type neuropathologic change (A3, B2, C2), Braak IV; cerebral amyloid angiopathy; small vessel disease.
**AZ107**	**MTG**	86	AD	M		COD: chest infection. Intermediate AD change (A2, B3, C1), Braak VI; Amygdala predominant Lewy body disease; deep small vessel disease; cerebral amyloid angiopathy.
**AZ108**	**MTG**	94	AD	F	11.5	COD: Alzheimer's disease. Consistent with Alzheimer's disease (NIA-AA score A3 B3 C2, high degree of AD change), LBD, amygdala predominant, cerebral amyloid angiopathy, hyaline arteriolosclerosis.
**AZ109**	**MTG**	90	AD	F	31	COD: end stage dementia. Consistent with Alzheimer's disease (NIA-AA score A3 B2 C1, intermediate AD change), cerebral amyloid angiopathy, hyaline arteriolosclerosis.
**AZ110**	**MTG**	86	AD	F	15	COD: severe dementia. Consistent with Alzheimer's disease (NIA-AA score A3 B3 C2, high AD change); hippocampal sclerosis with associated TDP-43 path; LBD, diffuse; focal cerebral amyloid angiopathy; small vessel disease.
**Avg**		83.4		47.6	17.5	

Table S2: Details of antibodies used for these studies.

Antigen	Species	Company	Catalogue	IHC (retrieval method)
**Primary antibody (clone)**				
**Amyloid beta (6F/3D)**	Mouse	Dako	M0872	1:100 (80 % formic acid)
**CD66B (G10F5)**	Mouse	Biolegend	305102	1:100 (Tris-EDTA pH 9.0)
**Collagen IV**	Rabbit	Abcam	ab6586	1:1000 (80 % Formic acid)
**Iba1 (poly)**	Goat	Abcam	ab5076	1:1000 (Tris-EDTA pH 9.0)
**L-ferritin (FTL/1386)**	Mouse	Abcam	ab218400	1:1000 (Tris-EDTA pH 9.0)
**GFAP (D1F4Q)**	Rabbit	Cell Signalling	12389	1:1000 (Tris-EDTA pH 9.0)
**GFAP (poly)**	Chicken	Abcam	ab4674	1:10000 (Tris-EDTA pH 9.0)
**Hemoglobin (poly)**	Goat	Abcam	ab19363	1:500 (80 % formic acid)
**Histone H3 (Cit R2/R8/R17)**	Rabbit	Abcam	ab5103	1:5000 (Tris-EDTA pH 9.0)
**HLA-DR/DP/DQ**	Mouse	Dako	M0746	1:500 (Tris-EDTA pH 9.0)
**MPO (poly)**	Rabbit	Dako	A0398	1:1000 (80 % formic acid)
**MPO (poly)**	Rabbit	Abcam	ab119106	1:500 (Tris-EDTA pH 9.0)
**MPO (poly)**	Goat	R&D	AF3667	1:500 (Tris-EDTA pH 9.0)
**MPO (poly)**	Goat	Santa Cruz	sc-16128	1:100 (Tris-EDTA pH 9.0)
**S100A8 (EPR3554)**	Rabbit	Abcam	ab92331	1:10000 (Tris-EDTA pH 9.0)
**NeuN (poly)**	Guinea pig	Millipore	ABN90	1:1000 (Tris-EDTA pH 9.0)
**P2YR12 (S16007D)**	Rat	Biolegend	848001	1:50 (Tris-EDTA pH 9.0)
**Tau (poly)**	Chicken	Abcam	ab75714	1:100 (80 % formic acid)
**Phospho-tau (AT8)**	Mouse	Invitrogen	MN1020	1:1000 (80 % formic acid)
**Biotinylated tomato lectin**	-	Vector Labs	B-1175-1	1:1000 (mouse only)
**Biotinylated UEA lectin**	-	Vector Labs	B-1065	1:1000 (human only)
**Secondary antibodies**				
**Anti-mouse Alexa 488**	Donkey	Life Tech	A-21202	1:500
**Anti-mouse Alexa 594**	Donkey	Life Tech	A-21203	1:500
**Anti-mouse Alexa 647**	Donkey	Life Tech	A-31571	1:500
**Anti-rabbit Alexa 594**	Donkey	Life Tech	A-32731	1:500
**Anti-rabbit Alexa 594**	Donkey	Life Tech	A-21207	1:500
**Anti-rabbit Alexa 647**	Donkey	Life Tech	A-31573	1:500
**Anti-goat Alexa 488**	Donkey	Life Tech	A-11055	1:500
**Anti-goat Alexa 594**	Donkey	Life Tech	A-11058	1:500
**Anti-goat Alexa 647**	Donkey	Life Tech	A-21447	1:500
**Anti-rat Alexa 488**	Donkey	Life Tech	A-21208	1:500
**Anti-guinea pig Alexa 488**	Donkey	Abcam	ab150187	1:500
**Anti-mouse IRDye-680RD**	Donkey	LiCOR	926-68072	1:500
**Anti-mouse IRDye-800CW**	Donkey	LiCOR	926-32212	1:500
**Anti-rabbit IRDye-680RD**	Donkey	LiCOR	926-68073	1:500
**Anti-rabbit IRDye-800CW**	Donkey	LiCOR	925-32213	1:500
**Anti-goat IRDye-680RD**	Donkey	LiCOR	926-68074	1:500
**Anti-goat IRDye-800CW**	Donkey	LiCOR	926-32214	1:500
**Streptavidin-Cy3**	-	Jackson IR	016-160-084	1 µg/mL
**Streptavidin-Cy5**	-	Jackson IR	016-160-074	1 µg/mL
